# Smoking behavior change and the risk of pneumonia hospitalization among smokers with diabetes mellitus

**DOI:** 10.1038/s41598-023-40658-9

**Published:** 2023-08-30

**Authors:** Dong-Woo Han, Wonyoung Jung, Kyu Na Lee, Kyungdo Han, Sei Won Lee, Dong Wook Shin

**Affiliations:** 1 Department of Pulmonology and Critical Care Medicine, Asan Medical Center, University of Ulsan College of Medicine, 88 Olympic-ro 43-gil, Songpa-gu, Seoul, 05505 Republic of Korea; 2 Department of Family Medicine and Supportive Care Center, Samsung Medical Center, Sungkyunkwan University School of Medicine, Seoul, Republic of Korea; 3 Department of Medicine, Sungkyunkwan University School of Medicine, Seoul, Republic of Korea; 4 Department of Statistics and Actuarial Science, Soongsil University, Seoul, Republic of Korea; 5 Department of Clinical Research Design and Evaluation, Samsung Advanced Institute for Health Science and Technology (SAIHST), Sungkyunkwan University, 81 Irwon-ro, Gangnam-gu, Seoul, 06351 Republic of Korea

**Keywords:** Immunology, Health care, Risk factors

## Abstract

Smoking patients with diabetes mellitus (DM) are at greater risk of developing pneumonia. How smoking behavior changes affect the risk of pneumonia hospitalization, however, remains unclear. Therefore, we investigated the association between smoking behavior change and the risk of pneumonia hospitalization in patients with DM. From January 1, 2009 and December 31, 2018, we investigated the association between smoking behavior change and the risk of pneumonia hospitalization in patients with DM. A total of 332,798 adult patients with DM from the Korean National Health Insurance System database who underwent health screening examination between 2009 and 2012, and were smokers at the first health examination were included. During a mean follow-up of 4.89 years, 14,598 (4.39%) incident pneumonia hospitalization cases were identified. Reducers had a slightly increased risk of pneumonia hospitalization (aHR 1.06, 95% CI 1.01–1.10) compared to sustainers. Quitters did not have a significant association with incidence of pneumonia hospitalization. However, increasers had 13% higher risk of pneumonia hospitalization (aHR 1.13, 95% CI 1.07–1.18), regardless of whether initial smoking was light, moderate, or heavy. Our study showed that an increase in smoking intensity was associated with an increased risk of pneumonia hospitalization in people with DM. However, a protective effect of smoking reduction or cessation on pneumonia risk was not demonstrated.

## Introduction

Diabetes mellitus (DM) is a global health problem. It affects over 500 million people worldwide, contributing to more than 10% of global mortality^1,2^. In Korea, the prevalence of DM has increased in accordance with the growth of the overweight or obese population^3^. Since poor glycemic control is linked with dysfunction of immune reaction^4^, people with DM are at increased risk of infection. Pneumonia is one of the most common infectious diseases in people with DM, and a leading infectious cause of death^5,6^.

Smoking is an established, modifiable risk factor of pneumonia^7^. Smoking induces inflammation on airways and impairs immune response including mucociliary clearance^7,8^, both of which elevate the risk of pneumonia. As DM and smoking are both risk factor of pneumonia, smoking patients with DM are at a greater risk of developing pneumonia. Despite of high risk of pneumonia, less than ten percent of people with DM are reported to completely quit smoking^9^.

In real world clinical practice, patients continuously change their smoking behavior; decreasing, quitting, and even increasing smoking amount often depends on circumstances, even if many physicians strongly recommend that their patients quit smoking. The relation between smoking behavior change and various diseases such as cardiovascular disease and cancer have already been reported^10–12^, and the benefit of smoking cessation and incidence of pneumonia in the general population was also studied^13^. However, the relation between smoking behavior changes and risk of pneumonia has not been studied in people with DM. Therefore, the present study aimed to investigate the association between smoking behavioral change and the risk of pneumonia hospitalization in the DM patient population.

## Methods

### Data source and study setting

We used the nationwide database provided by National Health Insurance Service (NHIS), covering 97% of the population in Korea. The NHIS recommends that all individuals aged ≥ 40 years and employers of all age among the insured, undergo a general health screening at least every two years^14^, which includes a standard questionnaire of past medical history, current medications, and lifestyle behaviors (smoking, drinking, and physical activity), anthropometric measurements (height, weight, body mass index, and blood pressure), and laboratory tests. In addition, medical treatment database (based on medical bills claimed by medical service providers for their medical expense claims) can be linked with the health examination database. Therefore, the NHIS retains an extensive health information dataset of the entire Korean population.

### Study population

**Figure 1:**
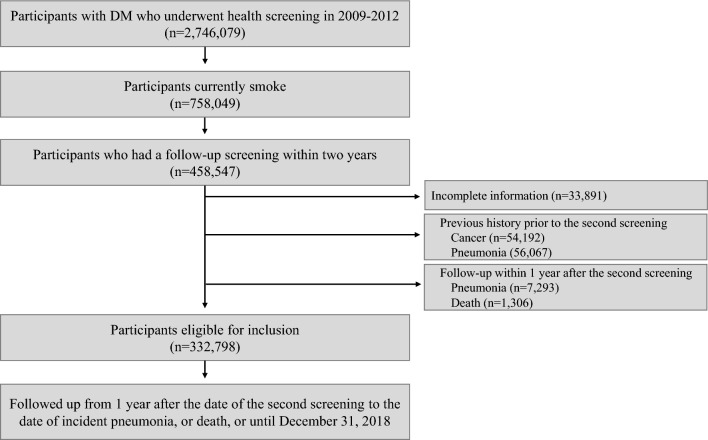
.

Among participants who underwent the health screening examination between 2009 and 2012 we identified 2,746,079 individuals with DM as follows: (1) fasting plasma glucose ≥ 126 mg/dL at the health screening or (2) a previous history of DM which was defined as International Classification of Disease (ICD)-10 code (E11–14) diagnosis with at least one claim per year for the prescription of antidiabetic agents before the health screening. This definition was based on the consensus of relevant findings widely used in previous studies^15,16^ ^4,5^. We excluded participants aged under 20 (n = 390), and then selected current smokers (n = 758,049) according to the definition of current smokers given by the World Health Organization^17^. Among them, 485,547 participants underwent the follow-up health examination within two years. We excluded those who had previously been diagnosed with any cancer (n = 54,192) or pneumonia (n = 56,067) before the second health screening and those with missing variables used in the study (n = 33,891). To reduce the effect of reverse causality, we applied a one-year lag time by excluding participants who were diagnosed with pneumonia (n = 7,293) and who died (n = 1,306) within one year after the second health examination period. Finally, we included a total of 332,798 participants (Fig. 1).

### Definition of change in amount of cigarette smoking

Information on smoking status and smoking behavior change was obtained from a self-administered questionnaire of the national health examination in the NHIS. The participants who answered that they were current smokers were asked about the average number of cigarettes per day and duration of smoking in years. At the first examination, participants were queried on the number of cigarettes smoked per day, and were grouped as (1) light smoker (< 10 cigarettes per day), (2) moderate smoker (10–19 cigarettes per day), and (3) heavy smoker (≥ 20 cigarettes per day). Then, s^7^tudy participants in each of the three groups were categorized into four subgroups by comparing the number of cigarettes per day between the first examination and the follow-up examination^18^: (1) quitters were those who quit smoking, (2) reducers were those who reduced the number of cigarettes by 20% or more, (3) sustainers were those who reduced the number of cigarettes by less than 20% or increased by less than 20%, and (4) increasers were those who increased the number of cigarettes by 20% or more.

### Study outcomes and follow-up

The primary endpoint of this study was pneumonia hospitalization, which was identified on the basis of the ICD-10 codes of J10.0, J11.0, and J12 to J18 administered for hospital admission. The cohort was followed from one year after the date of the second health examination to the date of pneumonia hospitalization, death, or until the end of the study period (December 31, 2018), whichever came first.

### Covariates

Household income was categorized into quartiles based on insurance premium levels (in Korea, insurance premiums are determined by income level), with those covered by Medical Aid (the poorest 3%) being merged into the lowest income quartile. Area of residence was dichotomized by rural and urban.

Alcohol drinking status classified participants into the following four groups according to the amount of alcohol consumed per day: (1) none, (2) mild (< 15 g of alcohol/day), (3) moderate (15–30 g of alcohol/day), and (4) heavy (≥ 30 g/day). Regular physical activity was defined as moderate physical activity for more than 30 min and more than 5 days per week during the past week. Body mass index was calculated using weight (kg) divided by height (m) in meters squared.

Comorbid medical conditions were assessed using comprehensive information regarding past medical history and clinical and pharmacy ICD-10 codes. Comorbidities including arterial hypertension (I10-I13 or I15), dyslipidemia (E78), chronic kidney disease (CKD; N18 or N19), interstitial lung disease [ILD, J84.0, J84.1, J84.8], asthma (J45 or J46), and chronic obstructive pulmonary disease [COPD, J43 or J44]) were included in our analysis.

For stratified analyses, DM patients were categorized considering DM severity by the duration of DM (newly-onset DM, DM of duration < 5 years, and DM of duration ≥ 5 years), the number of oral antidiabetic agents used (0, 1–2, and ≥ 3), and use of insulin.

### Statistical analysis

The baseline characteristics are presented as the mean ± standard deviation and number with percentage for categorical variables. The rates of pneumonia hospitalization were presented per 1,000 person-years. Cox proportional hazards regression analysis was conducted to evaluate the association between smoking behavior change and pneumonia hospitalization among individuals with DM. Model 2 was adjusted for age, sex, body mass index, household income, area of residence, duration of smoking, previous pack-years of smoking, alcohol consumption, regular physical activity, comorbidities (hypertension, dyslipidemia, CKD, and pulmonary diseases (defined as any of ILD, asthma, or COPD). Waist circumference, blood pressure, and laboratory findings was not included for adjustment to evade duplication with other covariates (body mass index, hypertension, dyslipidemia). At final model 3, as our study focused on DM population, DM-related variables including fasting glucose level, duration of DM, and insulin use were additionally adjusted compared to model 2.

Stratification analyses by age (< 65 years and ≥ 65 years), sex, smoking level at the first examination (light, moderate, or heavy smoker), pulmonary disease, duration of DM, the number of oral antidiabetic agents, and the use of insulin were performed to see the potentially different association of smoking behavior change with pneumonia hospitalization. The statistical analyses were performed using SAS version 9.4 (SAS Institute Inc., Cary, NC, USA). P values < 0.05 were considered statistically significant.

### Ethics statement

This study was approved by the Institutional Review Board of Samsung Medical Center (SMC 2022-07-072). Anonymized and de-identified information was used for analyses; therefore, informed consent was not required. The database is open to all researchers whose study protocols are approved by the official review committee.

## Results

### Baseline characteristics of study participants

Table 1 shows the baseline characteristics at the second examination according to the smoking behavior change (quitters, reducers, sustainers and increasers). Among the total 332,798 study participants (mean age 52.1 ± 11.0 years, male sex 95.7%), 8.3% were light smokers, 35.2% were moderate smokers, and 50.5% were heavy smokers in the first examination. Remarkably, 21.4% and 19.2% of total participants reduced and quit smoking during the two-year interval, respectively, while 16.7% increased their smoking amount. The sustainers and the increasers were more likely to be heavy smokers with longer durations of smoking. The quitters and the reducers tended to have high prevalence of hypertension, dyslipidemia, and CKD, accompanied with longer duration of DM, higher number of oral antidiabetic agents and more use of insulin than the sustainers.

**Table 1:** Baseline characteristics of study population according to the smoking behavior change.

	Total (n = 332,798)	Quitter (n = 61,941)	Reducer (n = 68,937)	Sustainer (n = 148,128)	Increaser (n = 53,792)	*P*-value
Age (years)	52.1 ± 11.0	54.5 ± 11.1	51.9 ± 11.2	51.4 ± 10.7	51.2 ± 11.1	< 0.001
Male sex	318,323 (95.7)	56,903 (91.9)	66,098 (95.9)	143,988 (97.2)	51,334 (95.4)	< 0.001
Income, quartile						< 0.001
Q1 (lowest)	61,419 (18.5)	11,453 (18.5)	13,184 (19.1)	26,562 (17.9)	10,220 (19.0)	
Q2	64,230 (19.3)	11,716 (18.9)	13,627 (19.8)	28,037 (18.9)	10,850 (20.2)	
Q3	97,932 (29.4)	16,879 (27.3)	20,177 (29.3)	44,687 (30.2)	16,189 (30.1)	
Q4 (highest)	109,217 (32.8)	21,893 (35.3)	21,949 (31.8)	48,842 (33.0)	16,533 (30.7)	
Urban residency	148,693 (44.7)	27,388 (44.2)	30,951 (44.9)	66,455 (44.9)	23,899 (44.4)	0.018
Anthropometrics						
Body mass index, kg/m^2^	24.8 ± 3.3	25.0 ± 3.2	24.8 ± 3.3	24.8 ± 3.3	24.9 ± 3.4	< 0.001
WC	86.0 ± 8.2	86.4 ± 8.0	86.0 ± 8.3	85.9 ± 8.2	86.0 ± 8.4	< 0.001
SBP	127.0 ± 51.7	127.4 ± 14.6	126.9 ± 14.7	126.8 ± 14.5	126.8 ± 14.8	< 0.001
DBP	79.0 ± 9.9	78.8 ± 9.9	79.0 ± 9.9	79.1 ± 9.9	79.1 ± 10.0	< 0.001
Smoking amount ^※^						< 0.001
Light (< 10 cigarettes/day)	27,514 (8.3)	4,445 (7.2)	14,578 (21.2)	5,365 (3.6)	3,126 (5.8)	
Moderate (10–19 cigarettes/day)	117,015 (35.2)	14,903 (24.1)	38,646 (56.1)	47,503 (32.1)	15,963 (29.7)	
Heavy (≥ 20 cigarettes/day)	168,182 (50.5)	22,506 (36.3)	15,713 (22.8)	95,260 (64.3)	34,703 (64.5)	
Duration of smoking, years						< 0.001
< 5	26,036 (7.8)	21,541 (34.8)	1,631 (2.4)	1,765 (1.2)	1,099 (2.0)	
5–9	9,248 (2.8)	1,859 (3.0)	2,360 (3.4)	3,290 (2.2)	1,739 (3.2)	
10–19	62,737 (18.9)	9,128 (14.7)	14,976 (21.7)	27,494 (18.6)	11,139 (20.7)	
20–29	102,995 (31.0)	13,083 (21.1)	21,765 (31.6)	50,622 (34.2)	17,525 (32.6)	
≥ 30	131,782 (39.6)	16,330 (26.4)	28,205 (40.9)	64,957 (43.9)	22,290 (41.4)	
Pack-years of smoking						< 0.001
< 10	77,666 (23.3)	29,834 (48.2)	21,421 (31.1)	18,730 (12.6)	7,681 (14.3)	
10–20	85,393 (25.7)	10,528 (17.0)	24,124 (35.0)	37,660 (25.4)	13,081 (24.3)	
20–30	73,088 (22.0)	8,600 (13.9)	12,570 (18.2)	40,024 (27.0)	11,894 (22.1)	
≥ 30	96,651 (29.0)	12,979 (21.0)	10,822 (15.7)	51,714 (34.9)	21,136 (39.3)	
Alcohol consumption						< 0.001
None	94,471 (28.4)	25,743 (41.6)	18,533 (26.9)	36,569 (24.7)	13,626 (25.3)	
Mild (< 15 g/day)	105,870 (31.8)	18,699 (30.2)	24,406 (35.4)	46,486 (31.4)	16,279 (30.3)	
Moderate (15–30 g/day)	72,644 (21.8)	9,897 (16.0)	15,041 (21.8)	35,498 (24.0)	12,208 (22.7)	
Heavy (≥ 30 g/day)	59,813 (18.0)	7,602 (12.3)	10,957 (15.9)	29,575 (20.0)	11,679 (21.7)	
Regular physical activity*						< 0.001
None	263,827 (79.3)	46,905 (75.7)	54,226 (78,7)	119,228 (80.5)	43,468 (80.8)	
Moderate or vigorous	50,819 (15.3)	10,871 (17.6)	10,945 (15,9)	21,385 (14.4)	7,618 (14.2)	
Moderate and vigorous	18,152 (5.5)	4,165 (6.7)	3,766 (5.5)	7,515 (5.1)	2,706 (5.0)	
Comorbidity^†^						
ILD	326 (0.1)	92 (0.2)	67 (0.1)	128 (0.1)	39 (0.1)	< 0.001
Asthma	40,454 (12.2)	8,913 (13.1)	8,371 (12.1)	16,733 (11.3)	6,437 (12.0)	< 0.001
COPD	36,431 (11.0)	6,093 (13.1)	7,423 (10.8)	15,224 (10.3)	5,691 (10.6)	< 0.001
Hypertension	157,706 (47.4)	32,348 (52.2)	32,538 (47.2)	68,252 (46.1)	24,568 (45.7)	< 0.001
Dyslipidemia	129,063 (38.8)	26,810 (43.3)	26,330 (38.2)	55,652 (37.6)	20,271 (37.7)	< 0.001
CKD	16,553 (5.0)	4,238 (6.8)	3,524 (5.1)	6,410 (4.3)	2,381 (4.4)	< 0.001
Laboratory findings						
Fasting glucose	138.0 ± 51.7	138.4 ± 50.7	137.3 ± 51.3	137.6 ± 51.4	139.3 ± 54.0	< 0.001
Cholesterol	193.0 ± 44.0	191.0 ± 47.2	192.6 ± 44.2	193.8 ± 43.3	193.4 ± 41.8	< 0.001
HDL-C	49.6 ± 18.4	50.0 ± 16.2	49.5 ± 25.8	49.5 ± 15.8	49.7 ± 15.7	< 0.001
LDL-C	107.6 ± 47.3	107.1 ± 45.7	107.4 ± 49.3	108.1 ± 47.6	107.4 ± 45.5	< 0.001
eGFR	92.3 ± 48.5	90.1 ± 49.9	92.3 ± 47.0	93.0 ± 48.7	93.2 ± 47.9	< 0.001
Duration of DM, years						< 0.001
New	145,182 (43.6)	22,530 (36.4)	30,496 (44.2)	67,823 (45.8)	24,333 (45.2)	
< 5	89,396 (26.9)	18,105 (29.2)	18,391 (26.7)	38,747 (26.2)	14,153 (26.3)	
≥ 5	98,220 (29.5)	21,306 (34.4)	20,050 (29.1)	21,558 (28.1)	15,306 (28.5)	
Number of antidiabetic agents						< 0.001
None	161,006 (48.4)	25,317 (40.9)	33,966 (49.3)	74,771 (50.5)	26,952 (50.1)	
1–2	120,972 (36.4)	25,490 (41.2)	24,597 (35.7)	52,022 (35.1)	18,863 (35.1)	
≥ 3	50,820 (15.3)	11,134 (18.0)	10,374 (15.1)	21,335 (14.4)	7,977 (14.8)	
Use of Insulin	17,580 (5.3)	4,445 (7.2)	3,623 (5.3)	6,840 (4.6)	2,672 (5.0)	

Data are expressed as mean ± standard deviation or number (%). WC, waist circumference; SBP, systolic blood pressure; DBP, diastolic blood pressure; ILD, interstitial lung disease; COPD, chronic obstructive pulmonary disease; CKD, chronic kidney disease; HDL-C, high-density lipoprotein cholesterol; LDL-C, low-density lipoprotein cholesterol; eGFR, estimated glomerular filtration rate; DM, diabetes mellitus.

*Regular physical activity was defined as > 30 min of moderate physical activity at least 5 times per week or > 20 min of vigorous physical activity at least 3 times per week.

^†^Comorbidities were based on claims data before the screening date and health screening results.

^※^Measured at the first examination.

### Incidence of pneumonia hospitalization according to smoking behavior change among people with DM

During the mean follow-up of 4.89 years, 14,598 (4.39%) incident pneumonia hospitalization cases were identified. With adjustment for social and clinical parameters, the reducers had a slightly increased risk of pneumonia hospitalization (aHR 1.06, 95% CI 1.01–1.10), compared to sustainers. Quitters did not have a significant association with incidence of pneumonia hospitalization (aHR 0.97, 95% CI 0.93–1.01). However, the increasers had 13% higher risk of pneumonia hospitalization (aHR 1.13, 95% CI 1.07–1.18, respectively). This significant increase in pneumonia hospitalization risk in the increasers was consistently found regardless of smoking amount and pack-years of smoking (Table 2).

**Table 2:** Associations between changes in cigarette smoking intensity and hospitalization for pneumonia in diabetes mellitus patients.

Smoking behavior		Subjects (N)	Events (n)	Hospitalization rate (per 1,000 PYs)	Model 1 (crude) HR (95% CI)	Model 2 aHR (95% CI)	Model 3 aHR (95% CI)
In 2009	In 2012						
All current smokers	Quitter	61,941	3,097	10.2	1.24 (1.19–1.30)	0.98 (0.94–1.03)	0.97 (0.93–1.01)
	Reducer	68,937	3,134	9.3	1.13 (1.08–1.18)	1.06 (1.01–1.11)	1.06 (1.01–1.10)
	Sustainer	148,128	5,952	8.2	1 (Ref.)	1 (Ref.)	1 (Ref.)
	Increaser	53,792	2,415	9.2	1.13 (1.08–1.18)	1.13 (1.08–1.19)	1.13 (1.07–1.18)
Smoking amount							
Light smokers^†^ (n = 27,514)	Quitter	11,219	596	11.1	1.04 (0.90–1.20)	1.02 (0.88–1.18)	1.01 (0.88–1.17)
	Reducer	3,291	210	13.5	1.26 (1.06–1.51)	1.15 (0.96–1.38)	1.16 (0.97–1.39)
	Sustainer	5,352	273	10.7	1 (Ref.)	1 (Ref.)	1 (Ref.)
	Increaser	11,550	627	11.4	1.07 (0.92–1.23)	1.20 (1.04–1.38)	1.20 (1.05–1.39)
Moderate smokers^†^ (n = 117,015)	Quitter	24,000	1,087	9.3	1.24 (1.15–1.33)	0.99 (0.92–1.07)	0.97 (0.90–1.05)
	Reducer	19,422	795	8.4	1.12 (1.03–1.22)	0.96 (0.88–1.04)	0.95 (0.87–1.03)
	Sustainer	47,393	1,722	7.5	1 (Ref.)	1 (Ref.)	1 (Ref.)
	Increaser	30,096	1,230	8.4	1.13 (1.05–1.21)	1.20 (1.11–1.29)	1.18 (1.09–1.27)
Heavy smokers^†^ (n = 168,182)	Quitter	26,722	1,414	10.7	1.26 (1.19–1.34)	1.02 (0.96–1.09)	1.00 (0.94–1.07)
	Reducer	46,224	2,129	9.4	1.11 (1.06–1.17)	1.09 (1.03–1.15)	1.09 (1.03–1.15)
	Sustainer	95,383	3,957	8.4	1 (Ref.)	1 (Ref.)	1 (Ref.)
	Increaser	12,146	558	9.3	1.10 (1.01–1.20)	1.25 (1.14–1.36)	1.22 (1.12–1.33)
Pack-years of smoking							
< 10^†^ (n = 77,666)	Quitter	29,834	1524	10.51	2.24 (2.01–2.49)	1.05 (0.94–1.17)	1.02 (0.92–1.14)
	Reducer	21,421	666	6.3656	1.36 (1.20–1.53)	1.00 (0.88–1.13)	0.99 (0.88–1.12)
	Sustainer	18,730	425	4.6732	1 (Ref.)	1 (Ref.)	1 (Ref.)
	Increaser	7,681	221	5.9752	1.28 (1.09–1.51)	1.08 (0.92–1.27)	1.07 (0.91–1.26)
10–20 (n = 85,393)	Quitter	10,528	337	6.5102	1.23 (1.09–1.40)	0.91 (0.80–1.03)	0.90 (0.79–1.02)
	Reducer	24,124	954	8.0699	1.53 (1.40–1.67)	1.11 (1.01–1.21)	1.10 (1.01–1.20)
	Sustainer	37,660	976	5.2758	1 (Ref.)	1 (Ref.)	1 (Ref.)
	Increaser	13,081	388	6.1065	1.16 (1.03–1.31)	1.13 (1.00–1.27)	1.13 (1.00–1.27)
20–30 (n = 73,088)	Quitter	8,600	338	7.9336	1.17 (1.04–1.32)	0.92 (0.82–1.04)	0.90 (0.80–1.01)
	Reducer	12,570	757	12.3409	1.83 (1.67–2.00)	1.18 (1.08–1.29)	1.16 (1.06–1.27)
	Sustainer	40,024	1325	6.7444	1 (Ref.)	1 (Ref.)	1 (Ref.)
	Increaser	11,894	448	7.712	1.14 (1.03–1.27)	1.10 (0.98–1.22)	1.10 (0.99–1.22)
≥ 30 (n = 96,651)	Quitter	12,979	898	14.0209	1.09 (1.02–1.18)	0.97 (0.90–1.04)	0.95 (0.88–1.02)
	Reducer	10,822	757	14.3107	1.12 (1.03–1.21)	1.13 (1.04–1.22)	1.12 (1.03–1.21)
	Sustainer	51,714	3226	12.775	1 (Ref.)	1 (Ref.)	1 (Ref.)
	Increaser	21,136	1358	13.2399	1.04 (0.97–1.11)	1.13 (1.06–1.20)	1.12 (1.05–1.19)

HR, hazard ratio; CI, confidence interval.

*Quitter, those who quit smoking; Reducer, those who reduced the number of cigarettes by 20% or more; Sustainer, those who reduced or increased the number of cigarettes by 20%; Increaser, those who increased the number of cigarettes by 20% or more.

^†^Light smokers, < 10 cigarettes/day; Moderate smokers, 10–19 cigarettes/day; Heavy smokers, ≥ 20 cigarettes/day.

Model 1: Unadjusted.

Model 2: adjusted for age, sex, body mass index, household income, area of residence, duration of smoking, alcohol consumption, physical activity, and comorbidities (hypertension, dyslipidemia, chronic kidney disease, and pulmonary diseases).

Model 3: Model 2 + adjusted for fasting glucose level, duration of diabetes mellitus, and use of insulin.

### Stratified analyses

After stratified analyses according to pulmonary diseases (Table 3), the association between smoking behavior change and the risk of pneumonia hospitalization was more prominent for participants without underlying pulmonary disease (P for interaction = 0.007), with 8% lower risk of pneumonia in quitters without underlying pulmonary diseases (aHR 0.92, 95% CI 0.87–0.97) compared to no lower risk for quitters with underlying pulmonary diseases (aHR 1.05, 95% CI 0.97–1.13). There was no significant interaction for other stratification variables.

**Table 3:** Associations between changes in cigarette smoking intensity and hospitalization for pneumonia according to age, sex, pulmonary disease, duration of diabetes mellitus, and the number of antidiabetic agents in diabetes mellitus patients.

			Pneumonia hospitalization			
			Subjects (N)	Events (n)	Hospitalization rate (per 1,000 PYs)	Model 3 aHR (95% CI)
Age	< 65	Quitter	50,468	1,505	6.02	0.98 (0.92–1.04)
		Reducer	59,826	1,740	5.90	1.06 (1.00–1.12)
		Sustainer	131,869	3,606	5.56	1 (Ref.)
		Increaser	47,515	1,462	6.29	1.17 (1.10–1.25)
	≥ 65	Quitter	11,473	1,592	29.76	0.95 (0.89–1.01)
		Reducer	9,111	1,394	33.00	1.05 (0.98–1.12)
		Sustainer	16,259	2,346	30.84	1 (Ref.)
		Increaser	6,277	953	33.23	1.06 (0.98–1.14)
	P for interaction					0.208
Sex	Male	Quitter	56,903	2,797	10.00	0.97 (0.93–1.02)
		Reducer	66,098	2,966	9.16	1.06 (1.02–1.11)
		Sustainer	143,988	5,700	8.08	1 (Ref.)
		Increaser	51,334	2,256	9.04	1.13 (1.08–1.19)
	Female	Quitter	5,038	300	12.64	0.90 (0.76–1.06)
		Reducer	2,839	168	12.55	0.90 (0.74–1.10)
		Sustainer	4,140	252	12.86	1 (Ref.)
		Increaser	2,458	159	13.70	1.05 (0.86–1.27)
	P for interaction					0.460
Smoking levels at the first examination	Light	Quitter	11,219	596	11.12	1.01(0.88–1.17)
		Reducer	3,291	210	13.49	1.16(0.97–1.39)
		Sustainer	5,352	273	10.68	1(Ref.)
		Increaser	11,550	627	11.38	1.21(1.05–1.39)
	Moderate	Quitter	24,000	1,087	9.27	0.97(0.90–1.05)
		Reducer	19,422	795	8.40	0.95(0.87–1.03)
		Sustainer	47,393	1,722	7.48	1(Ref.)
		Increaser	30,096	1,230	8.42	1.18(1.09–1.27)
	Heavy	Quitter	26,722	1,414	10.67	1.00(0.94–1.07)
		Reducer	46,224	2,129	9.39	1.09(1.03–1.15)
		Sustainer	95,383	3,957	8.43	1(Ref.)
		Increaser	12,146	558	9.29	1.22(1.12–1.34)
	P for interaction					0.177
Pulmonary disease	No	Quitter	47,917	1,853	7.81	0.92 (0.87–0.97)
		Reducer	55,736	2,071	7.54	1.05 (1.00–1.11)
		Sustainer	121,208	4,042	6.77	1 (Ref.)
		Increaser	43,615	1,628	7.63	1.15 (1.08–1.21)
	Yes	Quitter	14,024	1,244	18.77	1.05 (0.97–1.13)
		Reducer	13,201	1,063	17.03	1.06 (0.99–1.15)
		Sustainer	26,920	1,910	14.89	1 (Ref.)
		Increaser	10,177	787	16.40	1.09 (1.00–1.18)
	P for interaction					0.007
Duration of DM, years	New onset	Quitter	31,194	995	6.55	0.95 (0.88–1.02)
		Reducer	38,948	1,103	5.85	1.02 (0.95–1.10)
		Sustainer	85,174	2,249	5.47	1 (Ref.)
		Increaser	30,454	885	6.05	1.11 (1.03–1.20)
	< 5	Quitter	15,094	850	11.19	0.98 (0.90–1.17)
		Reducer	15,547	881	11.26	1.09 (1.01–1.19)
		Sustainer	33,376	1,620	9.60	1 (Ref.)
		Increaser	12,451	674	10.83	1.14 (1.04–1.25)
	≥ 5	Quitter	15,653	1,252	16.56	0.98 (0.91–1.05)
		Reducer	14,442	1,150	16.34	1.06 (0.99–1.14)
		Sustainer	29,578	2,083	16.34	1 (Ref.)
		Increaser	10,887	856	16.28	1.14 (1.05–1.23)
	P for interaction					0.953
Number of oral antidiabetic agents	None	Quitter	25,317	847	6.90	0.95 (0.88–1.03)
		Reducer	33,966	1,034	6.30	1.04 (0.97–1.12)
		Sustainer	74,771	2,060	5.72	1 (Ref.)
		Increaser	26,952	843	6.52	1.13 (1.04–1.22)
	1–2	Quitter	25,490	1,488	11.76	1.00 (0.94–1.07)
		Reducer	24,597	1,389	11.34	1.07 (1.00–1.14)
		Sustainer	52,022	2,574	9.87	1 (Ref.)
		Increaser	18,863	1,050	11.24	1.14 (1.07–1.23)
	≥ 3	Quitter	11,134	762	14.08	0.92 (0.84–1.01)
		Reducer	10,374	711	14.12	1.05 (0.96–1.15)
		Sustainer	21,335	1,318	12.68	1 (Ref.)
		Increaser	7,977	522	13.57	1.09 (0.98–1.20)
	P for interaction					0.833
Use of insulin	No	Quitter	57,496	2,617	9.27	0.97 (0.92–1.01)
		Reducer	65,314	2,752	8.60	1.05 (1.00–1.10)
		Sustainer	141,288	5,312	7.68	1 (Ref.)
		Increaser	51,120	2,140	8.61	1.13 (1.07–1.19)
	Yes	Quitter	4,445	480	22.69	0.97 (0.86–1.09)
		Reducer	3,623	382	22.21	1.08 (0.95–1.23)
		Sustainer	6,840	640	19.40	1 (Ref.)
		Increaser	2,672	275	21.66	1.11 (0.96–1.28)
	P for interaction					0.959

HR, hazard ratio; CI, confidence interval.

*Quitter, those who quit smoking; Reducer, those who reduced the number of cigarettes by 20% or more; Sustainer, those who reduced or increased the number of cigarettes by less than 20%; Increaser, those who increased the number of cigarettes by 20% or more; Pulmonary disease includes interstitial lung disease, asthma, or chronic obstructive pulmonary disease.

Model 3: adjusted for age, sex, body mass index, household income, area of residence, duration of smoking, alcohol consumption, physical activity, comorbidities (hypertension, dyslipidemia, chronic kidney disease, and pulmonary diseases), fasting glucose level, duration of diabetes mellitus, and use of insulin.

## Discussion

To the best of our knowledge, this is the first study to investigate the association between smoking behavior change and the risk of pneumonia hospitalization in the DM population. As we analyzed the nationwide, population-based, representative cohort by using data from the NHIS database, our study minimized selection bias and follow-up loss. We demonstrated that increased smoking in patients with DM is associated with 13% higher risk of pneumonia hospitalization. The benefit of reducing or quitting smoking was not demonstrated across the whole population, yet the benefit of quitting was noted in those without comorbid pulmonary diseases.

In this study, the prevalence of smoking at baseline was 27.6%, which is similar to the that of the general population in Korea (27.3%)^19^. During the two-year period, 21.4% of these smokers quit smoking and 19.2% reduced their smoking, whereas 16.7% increased smoking amount. While smoking cessation remains a major lifestyle modification to prevent complications in DM, our findings suggest that smoking cessation is not well-performed in real-world management of DM patients^12^.

Consistent with previous meta-analysis^7^, an increase in smoking in patients with DM is associated with 13% higher risk of pneumonia hospitalization. Tobacco smoking is well known to induce pneumonia in multifactorial ways. Smoking contributes to the pathogenesis of pneumonia by (1) structural impairment^20^, (2) disrupting innate and adaptive immune response^21^, and (3) modifying airway microbiome^22^. Due to this alteration of the defense system, smokers are known to be more susceptible to pneumonia and a dose–response relationship has been reported to exist between smoking amount and risk of pneumonia^8^. As smokers and patients with DM are more susceptible to developing pneumonia, our study provided a clear message that at least increases in smoking amount in patients with DM should be avoided and smoking should cease, especially in those without chronic pulmonary disease.

Notably, we observed that smoking reduction was associated with 6% increased risk of pneumonia hospitalization in people with DM. Numerous studies have demonstrated that both smoking reduction and cessation are related to a protective effect against pneumonia incidence in the general population^7,13^, which is inconsistent with our finding. Identifying the exact mechanism is beyond the scope of this study, but there are several possible explanations. First, residual inflammation from smoking can persist for a considerable period even after smoking cessation. It takes more than three years for the function of bronchial epithelium to recover in COPD^23,24^. Another study has shown that patients who quit smoking four or more years previous had lower incidence of pneumonia than patients who had quit smoking less than a year previous^8^. Since we observed smoking behavioral change for two years, a longer observational period may be required to examine the effect of smoking cessation. Second, the “sick-quitter effect” may be in play. “Sick” patients with overall worse condition and more comorbidities may have already quit or reduced their smoking amount. In our study, reducers and quitters had higher prevalence of hypertension, dyslipidemia, and CKD, accompanied with longer duration of DM, higher number of oral antidiabetic agents and more use of insulin, compared to sustainers.

In our study, quitters without comorbid pulmonary diseases had a slightly decreased pneumonia hospitalization risk, while such benefit was not noted in those with comorbid pulmonary diseases. Since previous studies showed people with pre-existing asthma^25^ or COPD^26^ had an increased risk of pneumonia incidence, the proinflammatory status from these underlying chronic pulmonary conditions could mitigate the protective effect of smoking cessation and reduction on developing pneumonia among people with DM^13^. Similarly, a Taiwanese study demonstrated that the effect of smoking on pulmonary tuberculosis was reduced in patients with DM^27^. Therefore, proinflammatory features of DM itself may have affected the protective effect of smoking behavioral change on pneumonia risk, which also may explain why the benefit of reducing or quitting smoking was not definite in our study.

With the strengths and some clinical implications of our study, there are, however, some limitations. First, generalization of our findings to other ethnicities or women would necessitate caution, since most of our study population consists of Korean males. Second, underestimation of smoking intensity is possible since smoking status was based on self-reported questionnaires. However, self-reported smoking behavior has been considered accurate with 87.5% sensitivity and 89.2% specificity according to a previous meta-analysis^28^. Third, we were not able to specify the etiology of pneumonia. However, considering the increased risk of all types of pneumonia related with immunomodulation by smoking, our result would be consistent regardless of subtypes of pneumonia^29^. Finally, because of limitation of NHI claim data, we could not check the severity of pneumonia and smoking status at the time of pneumonia hospitalization. Therefore, there were no certain criteria for pneumonia and hospitalization which can cause upcoding of pneumonia hospitalization depending on patients’ status including old age and socioeconomic status. In addition, smoking behavior change was classified depending on national health examination questionnaire of two times with interval, so patients’ smoking status could be different at the time of pneumonia hospitalization.

In conclusion, in patients with DM, the increase in smoking intensity was associated with an increased risk of pneumonia, but the protective effect of smoking reduction or cessation on pneumonia risk was not demonstrated, except for a slightly decreased risk of pneumonia hospitalization in quitters.
